# Single and Combined Effects of Pesticide Seed Dressings and Herbicides on Earthworms, Soil Microorganisms, and Litter Decomposition

**DOI:** 10.3389/fpls.2017.00215

**Published:** 2017-02-21

**Authors:** Willem Van Hoesel, Alexandra Tiefenbacher, Nina König, Verena M. Dorn, Julia F. Hagenguth, Urša Prah, Theresia Widhalm, Viktoria Wiklicky, Robert Koller, Michael Bonkowski, Jan Lagerlöf, Andreas Ratzenböck, Johann G. Zaller

**Affiliations:** ^1^Department of Integrative Biology and Biodiversity Research, Institute of Zoology, University of Natural Resources and Life Sciences ViennaVienna, Austria; ^2^Department of Terrestrial Ecology, Institute of Zoology, University of CologneCologne, Germany; ^3^Institute of Bio- and Geosciences, IBG-2: Plant Sciences, Forschungszentrum JülichJülich, Germany; ^4^Department of Ecology, Swedish University of Agricultural Sciences (SLU)Uppsala, Sweden; ^5^Austrian Agency for Health and Food Safety GmbH (AGES)Vienna, Austria

**Keywords:** agrochemicals, agroecology, neonicotinoids, non-target effects, pesticide, seed coatings, soil organisms, glyphosate-herbicide

## Abstract

Seed dressing, i.e., the treatment of crop seeds with insecticides and/or fungicides, aiming to protect seeds from pests and diseases, is widely used in conventional agriculture. During the growing season, those crop fields often receive additional broadband herbicide applications. However, despite this broad utilization, very little is known on potential side effects or interactions between these different pesticide classes on soil organisms. In a greenhouse pot experiment, we studied single and interactive effects of seed dressing of winter wheat (*Triticum aestivum* L. *var. Capo*) with neonicotinoid insecticides and/or strobilurin and triazolinthione fungicides and an additional one-time application of a glyphosate-based herbicide on the activity of earthworms, soil microorganisms, litter decomposition, and crop growth. To further address food-web interactions, earthworms were introduced to half of the experimental units as an additional experimental factor. Seed dressings significantly reduced the surface activity of earthworms with no difference whether insecticides or fungicides were used. Moreover, seed dressing effects on earthworm activity were intensified by herbicides (significant herbicide × seed dressing interaction). Neither seed dressings nor herbicide application affected litter decomposition, soil basal respiration, microbial biomass, or specific respiration. Seed dressing did also not affect wheat growth. We conclude that interactive effects on soil biota and processes of different pesticide classes should receive more attention in ecotoxicological research.

## Introduction

The prophylactic treatment of crop seeds with insecticides and/or fungicides, so called “seed dressing,” is very common in conventional agriculture, especially for wheat, oilseed rape, sugar beet, and maize (Krupinsky et al., 2002; Elbert et al., 2008). Many of the agrochemicals used for seed dressings act systemically, meaning that they will be distributed across the whole crop plant and potentially also released into the soil. Recently, neonicotinoid insecticides used for seed dressings have received increased attention because of their proved harm to insect pollinators (Gill et al., 2012; Whitehorn et al., 2012; Easton and Goulson, 2013; Pisa et al., 2015). Besides insecticides, various classes of fungicides are used for seed dressings. However, very little is known about their potential non-target effects. Due to the persistence of neonicotinoid pesticides in the soil of up to several years, non-target effects have been observed in various soil organisms inhabiting agroecosystems (Goulson, 2013; Köhler and Triebskorn, 2013; Chagnon et al., 2015; Pisa et al., 2015). Reports on effects of insecticide and fungicide seed dressings vary from stimulating Collembola surface activity (Zaller et al., 2016) to reducing collembolan reproduction (Alves et al., 2014), increasing numbers of protozoa and reducing plant decomposition rate (Zaller et al., 2016) to increasing earthworm mortality (Alves et al., 2013) or not influencing earthworm activity (Zaller et al., 2016).

Fields where dressed seeds are applied are often additionally treated with glyphosate-based herbicides, e.g., for pre-harvest desiccation (Carvalho et al., 2009). Although for decades this group of herbicides has been considered to be without harm toward non-target soil organisms, scientific evidence is mounting for adverse effects on symbiotic mycorrhizal fungi (Druille et al., 2013; Zaller et al., 2014), earthworms (Dalby et al., 1995; Morowati, 2000; Yasmin and D'souza, 2007; Piola et al., 2013; Pelosi et al., 2014; Gaupp-Berghausen et al., 2015), and soil microbial communities (Zabaloy et al., 2012; Imparato et al., 2016). However, the combined effects of different pesticide classes on soil organisms have received little attention (Yasmin and D'souza, 2007; Santos et al., 2011; Van Der Sluijs et al., 2015). Moreover, we are not aware of any study targeting the interactive effects of seed dressings and glyphosate-based herbicides on soil organisms and soil processes.

Microorganisms and soil fauna contribute to the decomposition of plant residues in agricultural fields, the mineralization of plant residues, and the recycling of plant nutrients (Berg, 2009; Paul, 2015). The soil macrofauna, especially vertically-burrowing earthworms, translocate plant material and plant seeds from the soil surface into deeper soil layers (Zaller and Saxler, 2007; Eisenhauer et al., 2008), creating hotspots of high microbial activity in deeper soil layers. Additionally, earthworms also feed on saprophytic fungi and other microorganisms driving decomposition (Scheu and Setälä, 2002; Curry and Schmidt, 2007). The resulting rate of litter decomposition will thus be an integrated effect of both earthworm and/or microbial activity (Hättenschwiler et al., 2005).

The aim of this study was to assess (i) to what extent pesticide seed dressings affect earthworm activity, soil microorganisms and litter decomposition, (ii) the existence of potential interactive effects between seed dressings and a glyphosate-herbicide application (i.e., cocktail effects) on these soil functional groups, and (iii) whether the presense of earthworms alters potential pesticide effects. We hypothesized, that insecticide and fungicide seed dressings would indirectly affect earthworms by reducing their microbial food sources. Fungicide seed dressing should exert negative effects on the fungal component of the soil microbial biomass (Merrington et al., 2002), while glyphosate-based herbicide application is assumed to decrease the overall activity of soil microorganisms (Sannino and Gianfreda, 2001; Zaller et al., 2014) and earthworms (Gaupp-Berghausen et al., 2015) and to increase respiration as a stress response of sensitive species (Zabaloy et al., 2012). Lastly, we expected non-target effects of two pesticide classes to be more severe than single applications.

## Materials and methods

### Experimental design

The experiment consisted of a full-factorial design including the factors Seed dressing (SD, 3 levels), Earthworms (EW, 2 levels), Herbicide application (Herbic, 2 levels), and their interactions; see below for details.

#### Experimental factors: seed dressings, earthworms, and herbicide application

We tested the effects of three types of seed dressings in this experiment: no seed dressing (treatment NO), seed dressing dominated by neonicotinoid insecticide and associated fungicides (treatment Insectic) and a fungicide seed dressing (treatment Fungic; Table 1). Each seed dressing treatment was replicated 10 times.

**Table 1:** **Overview of the seed dressing treatments and glyphosate-based herbicide used in the current experiment**.

**Treatment/Brand name**	**Active ingredient**	**Pesticide class**	**Chemical class**	**Conc. (g l^−1^)**	**Systemic?**
**INSECTICIDE SEED DRESSING**					
Gaucho 600FS edigo	lmidacloprid	Insecticide	Neonicotinoid	600	Yes
	Prothioconazole	Fungicide	Triazolinthione	100	Yes
Celest Extra 050FS	Difenoconazole	Fungicide	Conazole	25	No
	Fludioxonil	Fungicide	Pyrrole	25	No
**FUNGICIDE SEED DRESSING**					
EfA Universal	Fluoxastrobin	Fungicide	Strobilurin	75	No
	Prothioconazole	Fungicide	Triazolinthione	50	Yes
	Fluopyram	Fungicide	Pyridylethylamide	10	No
	Tebuconazole	Fungicide	Triazole	7.5	No
**G/YPHOSATE-BASED HERBICIDE**					
Roundup Lb Plus	Glyphosate	Herbicide	Organophosphate	360	Yes

Eight days after the seeding two adult specimens of *Lumbricus terrestris* L. per mesocosm were added to half of the mesocosms (total average earthworm fresh mass added across treatments: 7.5 ± 0.8 g mesocosm^−1^; treatment +EW); no earthworms were added to the other half of the mesocosms (treatment –EW). Each earthworm treatment was replicated 5 times.

Thirty-one days after seeding, a broadband glyphosate-based herbicide (Roundup Lb Plus; Monsanto Agrar Deutschland GmbH, Düsseldorf, Germany) was applied to half of the mesocosms (treatment +Herbic); no herbicide was applied to the other half of the mesocosms (treatment –Herbic). This resulted in five replicates of each of the above mentioned treatments after this stage.

### Experimental setup

The experiment was conducted in an experimental greenhouse at the University of Natural Resources and Life Sciences Vienna (BOKU), Austria (N48°14′12.4, E16°20′08.4). The 60 cylindrical mesocosms (diameter: 25 cm, height: 60 cm, volume: 30 l) were randomly placed in three double-rows each consisting of 2 × 10 mesocosms in east-west direction. The mesocosms were filled with a soil mixture consisting of a substrate mixture of 75% vol/vol haplic chernozem from an arable field of the BOKU Research Farm (Groß Enzersdorf, Austria) that was mixed with 1.4–2.2 mm quartz sand (general soil characteristics are: C:N ratio 17.15, pH 7.45 ± 0.02) and 25% commercial peat-free potting soil containing bark humus, wood fibers, and green waste compost, sand, and mineral (NPK) fertilizer. No soil sterilization was performed. All mesocosms were outfitted with a 20 cm high barrier of plastic sheet glued at the top of the mesocosm in order to prevent any organisms from escaping. Between October and December 2013, these mesocosms were used to test non-target effects of seed dressings on earthworms and Collembola and the soil decomposition processes by microorganisms (Zaller et al., 2016). After the termination of the previous experiment, mesocosms were kept in the greenhouse, watering was stopped and heating was kept at 20°C in order to induce a complete dry-out and defaunation of the soil. After 3 months, careful examinations did not show any signs of earthworm or Collembola activity in the pots. For the current experiment, the original treatments were retained: i.e., seed dressings and earthworm treatments were assigned to the same mesocosms than in the former experiment. The Collembola treatment of the former experiment was excluded in the current experiment; no Collembola activity was observed during the course of the current study.

Each mesocosm was sown with 18 seeds treated with pesticide seed dressings (Table 1) of winter wheat (*Triticum aestivum* L. *var. Capo*) placed in 1 cm depth in a consistent pattern. Seeding density corresponded to 367 seeds m^−2^ that is within the recommended seeding density for this variety. Seed material with these dressings is available for Austrian farmers and was provided by the Austrian Agency for Health and Food Security (AGES, Vienna, Austria).

Added earthworms were purchased at a local fishing equipment shop in Vienna (www.anglertreff.at). Earthworms were placed on the soil surface and buried themselves into the soil within several minutes. During the experiment, all mesocosms received 1 g of dried hay per week, placed at the top of the soil; the mesocosms that did not contain earthworms received the same amount of hay to ensure equal nutrient input.

Thirty-one days after seeding, Roundup Lb Plus was applied to wheat plants that were about 12 cm high at that time. Roundup Lb Plus contains 30.8% glyphosate as active ingredient; 486 g l^−1^ as isopropylamin salt (Table 1). This formulation is registered for use in arable crops, forestry, horticulture, viticulture, and private use (http://pmg.ages.at/export/PMG/PMG/web/reg/3393_901.html). We applied the herbicide as recommended on the manual of the spray bottle, so that all plants were covered with a mist. This resulted in a total of 1.47 ml m^−2^ that is 1.47 times the recommended application amount of 1 ml m^−2^ of this product. Mesocosms near the treated ones were protected by a plastic sheet. Plant death due to the herbicide application was observed about 7 days after spraying.

The current experiment lasted from March until June 2014, covering 97 days. The average air temperature during this period was 18 ± 2.4°C (mean ± standard deviation) at a relative humidity of 59.4 ± 29.5%; measured with data loggers placed 2 m above the greenhouse floor (Tinytag, Gemini Data Loggers, UK).

### Measurements

#### Earthworm activity, earthworm survival, and development

In order to assess surface activity of earthworms, the toothpick method was used (Zaller et al., 2014). Briefly, 12 regular wooden toothpicks (length: 6.5 cm) were randomly inserted vertically across the surface, with the tips slightly stuck in the ground. Earthworms foraging aboveground knock over or incline the toothpicks; the number of toothpicks differing from their original upright position thus indicates the above ground activity of earthworms. Toothpicks were inserted in the evening and assessed the following morning; this was regularly done once a week resulting in five assessments before herbicide application, and twice a week after the herbicide application (3–4 day interval) resulting in 16 assessments. Toothpicks were removed between sampling dates. For the analysis of earthworm activity three different categories of disturbance of the toothpicks were used: value 0.1 for slight disturbance, 0.5 for disturbance in which the toothpick was tilted more than or around 45° and 1 for those that were found horizontally on the surface. The number of the toothpicks within each category was multiplied with the category value and then summed and taken as an index measure of aboveground earthworm activity. Because we were interested to see if either the size or the number of earthworms is more responsive to our pesticide treatments, activity of earthworms was further expressed as number of toothpicks moved per g earthworm biomass (specific earthworm activity) and per number of earthworms (individual earthworm activity). After wheat harvesting, mesocosms were turned over and each mesocosm searched for earthworms by two persons for 8 min. Earthworms were counted, washed free of attached soil, carefully dried off on a paper towel, and collectively weighed per mesocosm.

#### Soil microbial biomass and activity

At the end of the experiment (97 days after seeding) five random soil samples per mesocosm were taken with a soil corer (diameter 1 cm, depth 5 cm) for the analysis of soil basal respiration, microbial biomass, and specific respiration. These samples were stored in polypropylene plastic bags, cooled at 5°C and express-mailed to the soil laboratory of the Department for Terrestrial Ecology at the University of Cologne, Germany, for analysis. Soil microbial biomass (C_*mic*_) was determined from a 3 g subsample of fresh soil samples. Microbial biomass was measured by substrate-induced respiration (Anderson, 1978) using an automated respirometer based on electrolytic O_2_ micro compensation (Scheu, 1992), as outlined in Beck et al. (1997). For basal respiration, the average O_2_ consumption rate of samples not amended with glucose was measured during 15–20 h after attachment of samples to the respirometer. Microbial specific respiration (qO_2_, μl O_2_ μg^−1^ C_*mic*_ h^−1^) was calculated as the quotient between basal respiration and microbial biomass.

#### Litter decomposition

Litter decomposition rate (k) and stabilization factor (S) were assessed using the tea bag method (Keuskamp et al., 2013) to assess the breakdown of labile of easily degradable and recalcitrant organic matter. In every mesocosm, we inserted 2 tea bags containing rooibos tea (Lipton, EAN: 87 22700 18843 8) and 2 containing green tea (Lipton, EAN: 87 22700 05552 5) at 8 cm depth. The tea bags were dried for 2 days at 55°C and weighed before insertion into the soil and were left in the soil for 84 days. Afterwards tea bags were excavated, cleaned from adhered soil particles, and dried for 3 days at 55°C and weighed. The decomposition rate (k) and the stabilization factor (S) was calculated after Keuskamp et al. (2013) considering the hydrolysable fraction of 0.842 g g^−1^ for green tea and of 0.552 g g^−1^ for rooibos tea. Green tea and rooibos tea have different decomposition rates meaning that rooibos tea decomposes slower and continues when labile material in green tea has already been consumed. The stabilization process begins during the decomposition of the labile fraction of organic material (Prescott, 2010). This method was already used in some other studies to examine non-target effects of glyphosate-based herbicides (Gaupp-Berghausen et al., 2015) and insecticide and fungicide seed dressings (Zaller et al., 2016).

In each mesocosm, soil moisture was measured using time domain reflectometry (Trime Pico 63/32 probe; IMKO, Micromodultechnik GmbH, Ettlingen, Germany). These measurements were taken once a week by inserting the 20 cm long probe in the center of each mesocosm.

#### Wheat growth and biomass production

Wheat height was recorded once a week on all plants per mesocosm by measuring their height from the soil surface to the tip of the longest leaf. Height measurement was stopped in mesocosms after the herbicide was applied on day 31 after seeding. On day 43 of the experiment, above ground biomass from all the mesocosms was harvested by cutting all wheat plants at the soil surface using scissors, then dried for 48 h at 55°C and weighed. The plant density per mesocosm on the day of herbicide spraying was on average 16.3 ± 1.3 plants mesocosm^−1^ and at the moment of final harvest of the remaining mesocosms 16.4 ± 1.1 plants mesocosm^−1^.

### Statistical analyses

All variables were tested for normality using P-P plots and homogeneity of variances using the Levene test and log transformed when necessary. Influence of seed dressing (SD) or herbicide application (Herbic) on earthworm surface activity (average moved toothpicks pot^−1^ day^−1^, cumulated moved toothpicks, activity per earthworm biomass) was tested using repeated measures analysis of variance (ANOVA). When Mauchly's Test of Sphericity indicated that the assumption of sphericity had been violated, the Greenhouse-Geisser correction was used. Three factorial analysis of variance (ANOVA) with the factors SD, Herbic or Earthworms and interactive effects among SD × Herbic, SD × Earthworms or Herbic × Earthworms was included in the statistical model to examine effects on litter decomposition, soil basal respiration, qCO_2_, microbial biomass, wheat growth, and wheat biomass as well as on earthworm numbers and biomass at the end of the experiment. In all analyses soil moisture content was used as a covariate. Statistical analyses were carried out using Minitab statistical software (Release 14, Minitab Corp., PA, USA).

## Results

**Figure 1:**
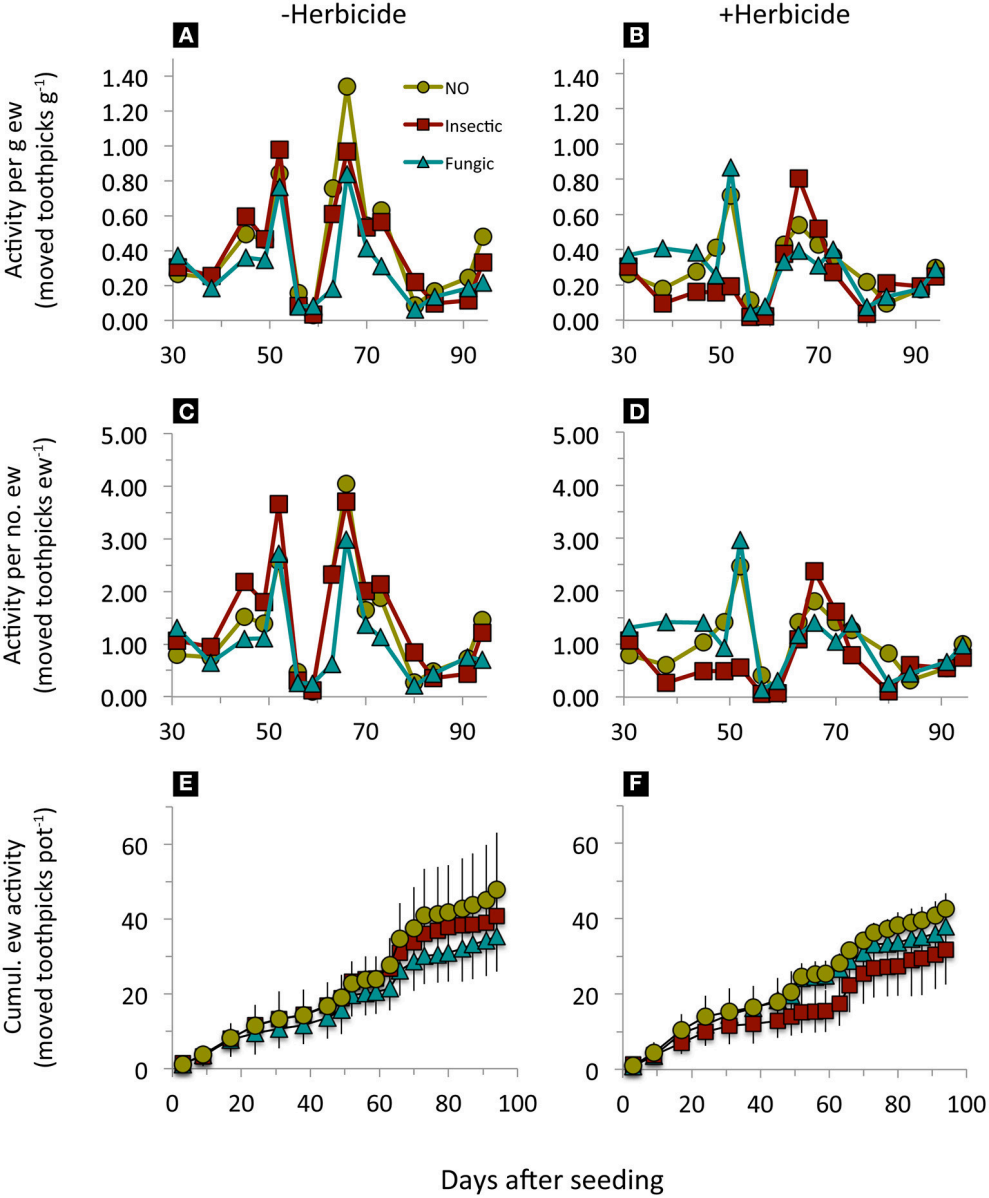
**Earthworm surface activity in mesocosms where winter wheat with different pesticide seed dressings was sown (NO, no seed dressing; insectic: neonicotinoid insecticide seed dressing; fungic: fungicide seed dressing) without (A,C,E) or with glyphosate-based herbicide application (B,D,F) at day 31**. Means, *n* = 5. Statistical results in Table 2.

Earthworm surface activity (per g earthworm, per number of earthworms, and cumulated surface activity) was significantly reduced by seed dressings compared to undressed seeds, regardless whether insecticide or fungicide seed dressings were used (Figure 1, Table 2). Across seed dressings, herbicide application reduced specific earthworm activity; while individual earthworm activity was affected by an interaction between seed dressings and herbicide application (Figure 1, Table 2). In our experiment, seed dressings reduced (cumulative) earthworm activity by 9.2% while herbicide application reduced it by 19.3%.

**Table 2:** **Statistical results testing the effects of seed dressings (SD), earthworms (EW) and glyphosate-herbicide application on earthworm surface activity, litter decomposition, soil microbial activity, wheat growth, and wheat biomass production in mesocosms**.

**Parameter**	**Seed Dressing (SD)**		**Earthworms (EW)**		**Herbicide (Herbic)**		***SD* × EW**		***SD* × Herbic**		**EW × Herbic**	
	***F***	***P***	***F***	***P***	***F***	***P***	***F***	***P***	***F***	***P***	***F***	***P***
EW surf. act., specific (toothp. g^−1^ EW)^rmA^	4.349	**0.025**	n.a.	n.a.	4.44	**0.046**	n.a.	n.a.	2.872	0.077	n.a.	n.a.
EW surf. act., specific (toothp. no^−1^ EW)^rmA^	2.776	0.083	n.a.	n.a.	3.571	0.071	n.a.	n.a.	4.769	**0.019**	n.a.	n.a.
EW surf. act., mean (toothpicks)^rmA^	4.011	**0.033**	n.a.	n.a.	2.619	0.120	n.a.	n.a.	0.440	0.649	n.a.	n.a.
EW cumul. surf. act. (toothpicks)^rmA^	3.742	**0.040**	n.a.	n.a.	0.116	0.736	n.a.	n.a.	2.422	0.112	n.a.	n.a.
Litter decomposition rate (k)	1.856	0.168	14.987	**<0.001**	0.001	0.971	0.252	0.092	1.159	0.323	0.416	0.522
Litter stabilization index (S)	1.410	0.254	14.463	**<0.001**	0.005	0.942	2.399	0.102	0.658	0.522	1.527	0.223
Soil basal respiration (1-Jg COr C g^−1^ h-1)	0.525	0.595	0.02	0.889	0.428	0.516	1.651	0.203	0.279	0.758	0.132	0.718
Soil microbial biomass (Cmic, I-Jg C g- 1)	1.527	0.228	1.522	0.224	0.183	0.671	0.032	0.969	1.356	0.268	0.046	0.831
Soil qC02 (μg C0_2_–C g- h^−1^ Cmic h^−1^)	0.880	0.164	0.2	0.657	1.069	0.307	0.751	0.478	2.036	0.142	0.219	0.642
Wheat height (cm)^rmA^	1.843	0.181	9.843	**0.005**	n.a.	n.a.	1.214	0.315	n.a.	n.a.	n.a.	n.a.
Wheat biomass (g)	0.668	0.517	4.925	**0.032**	n.a.	n.a.	0.377	0.688	n.a.	n.a.	n.a.	n.a.

*Significant effects in bold*.

*n.a. Not applicable; ^rmA^ analyzed with repeated measures ANOVA*.

At the end of the experiment we found 88.3% of the initially inserted adult earthworms and 59.6% of the initially inserted biomass of earthworms (Table 3). Neither the number, nor the biomass of retrieved adult earthworms at harvest was affected by seed dressings, herbicide applications or their interactions (adult earthworm numbers: *SD* – *F* = 1.142, *P* = 0.345; Herbic – *F* = 0.111, *P* = 0.744; SD × Herbic – *F* = 0.331, *P* = 0.723; adult earthworm biomass: *SD* – *F* = 1.352, *P* = 0.288; Herbic – *F* = 0.088, *P* = 0.770; *SD* × Herbic – *F* = 0.937, *P* = 0.414). Moreover, at the end of the experiment, all mesocosms contained many juvenile, initially not inserted earthworms. Mesocosms initially without earthworm inoculation contained at harvest 42.47 ± 19.02 juvenile individuals with 0.85 ± 0.41 g mesocosm^−1^, mesocosms with earthworm inoculation contained 42.17 ± 21.18 juvenile individuals with 5.46 ± 4.46 g mesocosm^−1^. Total earthworm numbers retrieved at harvest was neither affected by earthworm treatment (*F* = 0.583, *P* = 0.449), seed dressing (*F* = 1.249, *P* = 0.296), herbicide application (*F* = 0.722, *P* = 0.400) nor the interactions of these factors. Total biomass of earthworms at harvest was significantly lower in non-earthworm treatments (*F* = 33.253, *P* < 0.001) but not affected by seed dressing (*F* = 0.525, *P* = 0.595), herbicide (*F* = 0.713, *P* = 0.403) or their interactions.

**Table 3:** **Earthworm numbers and biomass (fresh mass) retrieved from mesocosms where winter wheat with seed dressings was sown or glyphosate-herbicide was applied**.

**Experimental factors**		**Adult earthworms (*L. terrestris*)**		**Juvenile unidentified, earthworms**	
**Seed dressing**	**Herbicide**	**Number**	**Biomass (g)**	**Number**	**Biomass (g)**
NO	No	1.6 ± 1.5	3.2 ± 3.6	38 ± 26	0.607 ± 0.411
	Yes	2.0 ± 1.2	5.4 ± 3.3	45 ± 21	1.008 ± 0.647
Insecticide	No	1.0 ± 1.4	3.0 ± 4.2	41 ± 18	0.840 ± 0.441
	Yes	2.0 ± 1.2	4.6 ± 3.8	38 ± 19	0.697 ± 0.399
Fungicide	No	2.2 ± 2.6	5.7 ± 7.2	45 ± 17	0.874 ± 0.329
	Yes	1.8 ± 2.2	4.9 ± 5.4	38 ± 26	0.918 ± 0.822

*Means ± st. dev., n = 10 for factor seed dressing; n = 5 when glyphosate was applied. No significant effects of seed dressings or herbicide application on earthworm numbers or biomass were observed*.

**Figure 2:**
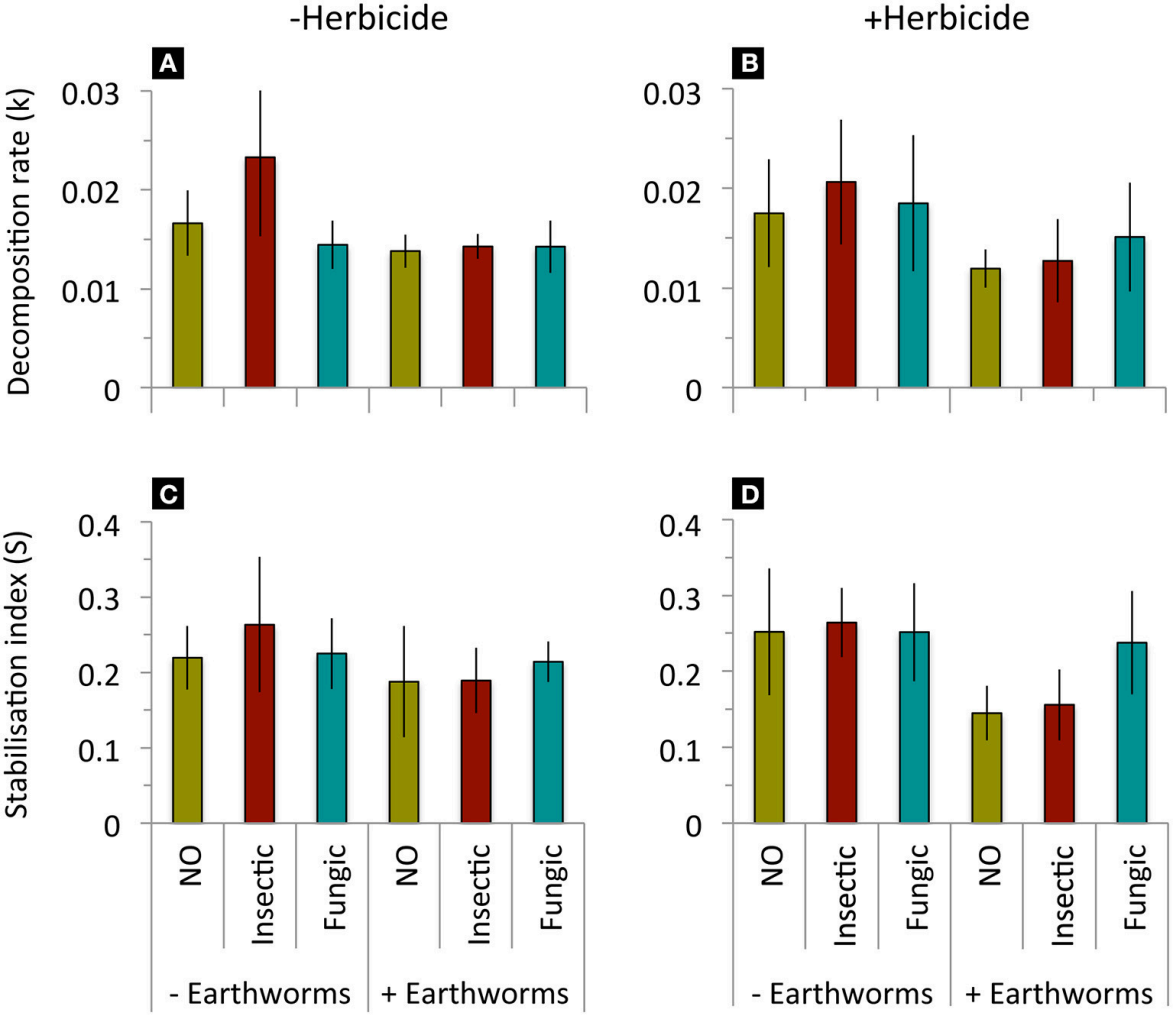
**Litter decomposition decomposition rate (k) and stabilization factor (S) in mesocosms without or with addition of *L. terrestris* earthworms, without (A,C) or with (B,D) glyphosate-based herbicide application**. Means ± st. dev., *n* = 10. Statistical results in Table 2.

**Figure 3:**
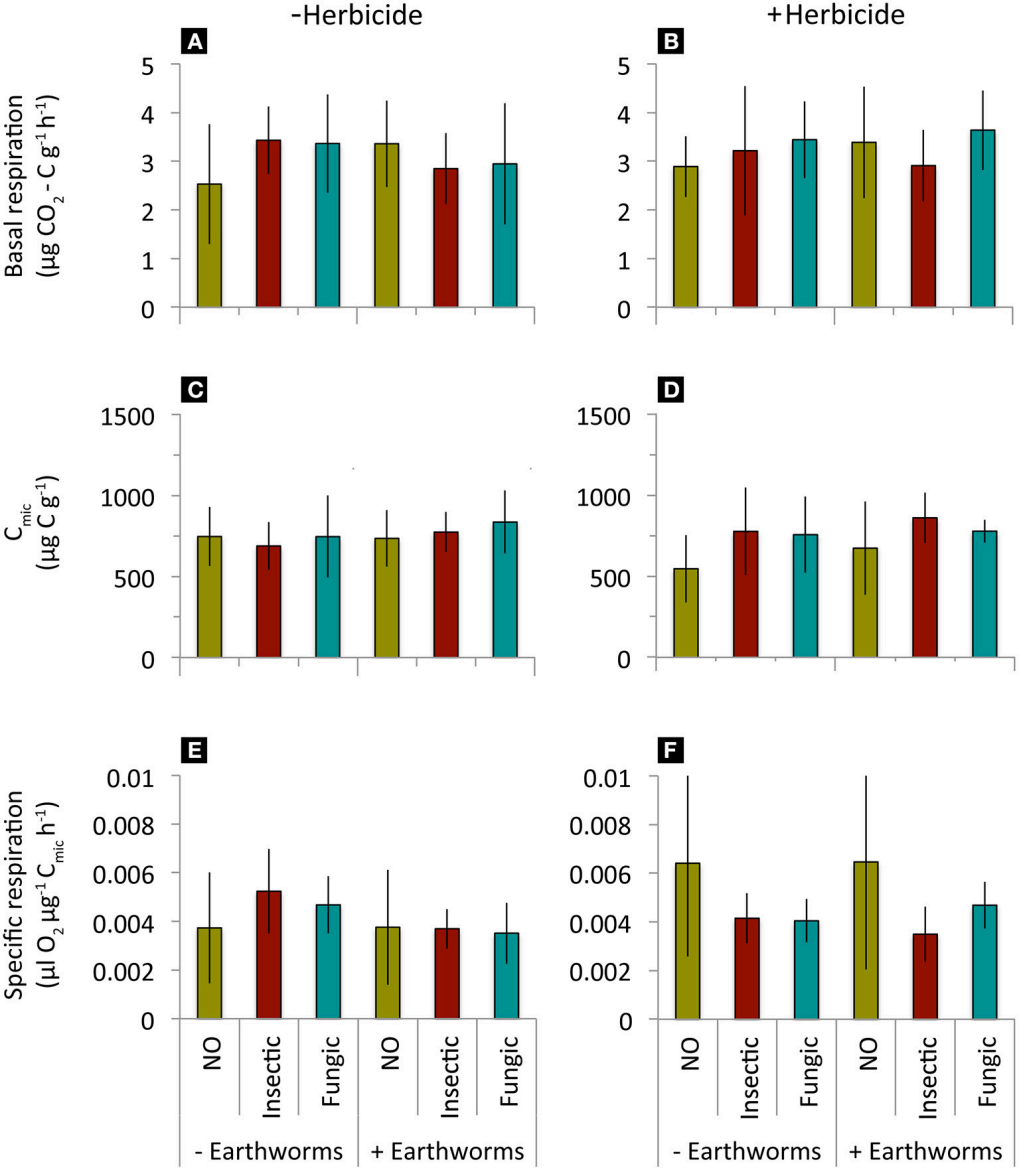
**Soil basal respiration, microbial biomass and specific respiration in mesocosms where winter wheat treated with different seed dressings (NO, no seed dressing; Insectic, neonicotinoid insecticide seed dressing; Fungic, fungicide seed dressing) were sown, without or with addition of *L. terrestris* earthworms and without (A,C,E) or with (B,D,F) glyphosate-herbicide application**. Means ± st. dev., *n* = 10. Statistical results in Table 2.

Litter decomposition rate (k) and stabilization factor (S) decreased when added *L. terrestris* earthworms were present, but were not affected by seed dressings, herbicide application or their interactions (Table 2, Figure 2). Soil basal respiration, microbial biomass, and specific respiration were neither affected by seed dressings, earthworm, herbicide application, or their interactions (Table 2, Figure 3).

**Figure 4:**
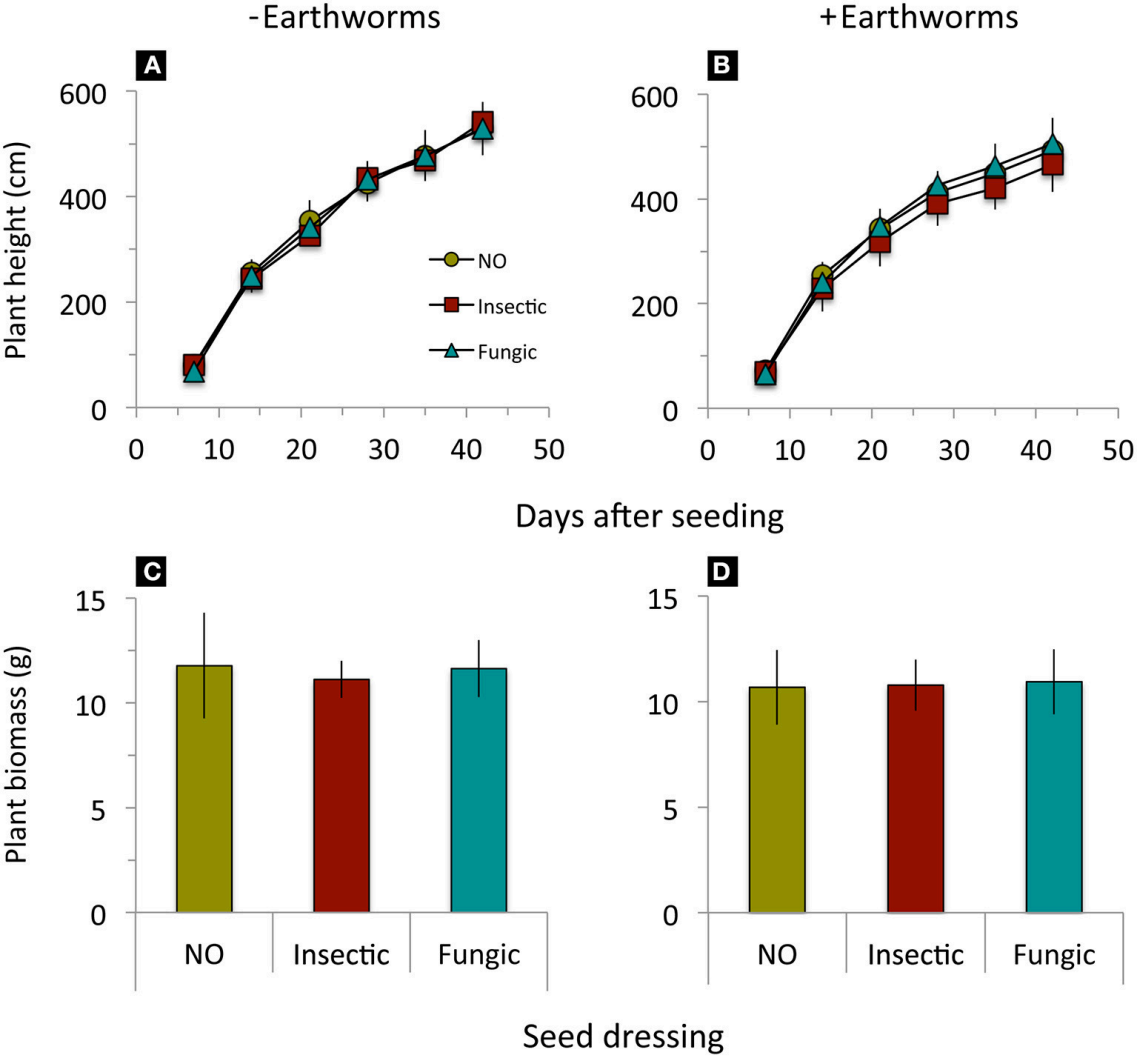
**Height growth and shoot biomass production of winter wheat treated with different seed dressings (NO, no seed dressing; Insectic, neonicotinoid insecticide seed dressing; Fungic, fungicide seed dressing) grown in mesocosms without (A,C) or with (B,D) addition of *L. terrestris* earthworms**. Means ± st. dev., *n* = 10. Statistical results in Table 2.

Wheat height and biomass production until 3 days before herbicide application were significantly reduced by earthworm activity but not affected by seed dressings nor an interaction between earthworms and seed dressings (Table 2, Figure 4).

## Discussion

To the best of our knowledge, this study is among the first studies addressing single and combined effects of different pesticide classes on soil organisms and soil processes. We aimed to mimic a typical farmland situation: wheat sown with pesticide treated seeds receiving an additional herbicide application later in the season. Our findings showed that seed dressings reduced earthworm activity regardless which pesticide class was used for seed treatment. Herbicide application itself reduced, and in interaction with seed dressings further decreased earthworm activity. Activity of soil microorganisms or litter decomposition appeared to be little affected by these pesticides.

### Seed dressing effects

The current study is an expansion of a previous one where the effect of seed dressings were studied on the activity of earthworms, Collembola, and soil microorganisms (Zaller et al., 2016). In the current experiment we additionally applied a herbicide treatment in order to test a common farmland situation. Our current findings are partly in contrast with our previous findings where no effects of seed dressings on earthworm activity and a reduced litter decomposition in response to seed dressings was observed (Zaller et al., 2016). We attribute the different outcomes to the following reasons. First, in the previous study seed dressings were applied for the first time, while in the current experiment by utilizing the mesocosms from the previous experiment, seed dressings were applied for a second time within 5 months. Studies have shown that pesticides from seed dressing application (at least with neonicotinoid insecticides) accumulate in soils which could have resulted in an increased impact on non-target organisms (Goulson, 2013). Second, the response of soil microorganisms and litter decomposition after a one-time application of pesticides (Zaller et al., 2016) suggest an initial sensitivity but a rapid adaptation of soil microorganisms to metabolize these substances (Griffiths et al., 2001; Liu et al., 2011; Cycon et al., 2013). Similar to the previous study, we found no effect of seed dressings on crop growth or biomass production. This might be due to a reduced pressure of pest insects or fungal diseases in our greenhouse experiment. However, even under field conditions the effect of seed dressing on yields appears to be negligible (Goulson, 2013; Budge et al., 2015; Furlan and Kreutzweiser, 2015).

There is good evidence that neonicotinoid insecticides directly affect earthworms (Dittbrenner et al., 2010, 2011). However, while we investigated actual pesticide formulations used by farmers, others investigated effects of the direct active ingredients. Significant loss in body mass of *L. terrestris* was observed with imidacloprid at concentrations ranging between 0.66 and 4.00 mg kg^−1^ soil after only 7–14 days of exposure (Dittbrenner et al., 2010, 2011). These sub-lethal effects occur well below the Predicted Environmental Concentration range of 0.33–0.66 mg kg^−1^ soil (Dittbrenner et al., 2010). Most likely, overall pesticide concentrations in soil were much lower in the current study with addition of only 16 treated seeds per mesocosm, however no data are available of the concentrations in the soils in the mesocosms used. We assume that earthworms perhaps also came in direct contact with the pesticides by feeding on the treated seeds (Milcu et al., 2006; Zaller and Saxler, 2007; Forey et al., 2011).

For other earthworm species than used in the current study, neonicotinoid insecticides resulted in an avoidance of treated soils (Dittbrenner et al., 2011, 2012), an altered burrowing activity (Capowiez et al., 2003, 2006; Capowiez and Bérard, 2006), DNA damage (Zang et al., 2000) or increased mortality (Tu et al., 2011). When comparing the acute toxicity of 24 insecticides on the earthworm species *E. fetida*, the neonicotinoid imidacloprid was listed in the category super toxic in both contact filter paper and soil toxicity bioassay tests (Wang et al., 2012a,b). However, earthworm responses to pesticides have been shown to be species-specific and the reaction of one species precludes a serious assessment across all earthworms (Pelosi et al., 2014; Pisa et al., 2015). Earthworm species also differ in their response to different pesticide classes: species feeding on the soil surface, are more affected by pesticides applied aboveground than those feeding deeper in the soil (Pelosi et al., 2014). Besides insecticides also fungicides had detrimental effects on earthworms (Jänsch et al., 2006). However, in comparison with herbicide and fungicides, insecticides show a more negative effect on three earthworm species (*Allobophora chlorotica, Lumbricus castaneus, L. terrestris)* (Pelosi et al., 2013).

Studies testing non-target effects of fungicide classes used in our seed dressings are very rare. A triazole fungicide application resulted in a negative impact on the epidermic cells of *E. fetida* earthworms (Gao et al., 2013), however it is unclear whether there was a similar mode of action in our earthworm species. Clearly, there is a great demand for more studies in this subject.

### Herbicide effects

The reduction in earthworm activity after application of glyphosate-based herbicide is in accordance with recent findings studying the same earthworm species (Gaupp-Berghausen et al., 2015) although a lower dosage was used in the current experiment. After the herbicide application, the seed dressings further reduced earthworm activity, indicating possible synergistic effects of these different pesticide classes. In other studies a glyphosate-based herbicide also reduced reproduction (Casabe et al., 2007; Gaupp-Berghausen et al., 2015) and led to decreased growth and survival (Eijsackers et al., 2005). Another study shows that glyphosate herbicide application resulted in a high percentage (50%) of lethargic *Lumbricus* sp., while the combined effect with a pesticide resulted in increased mortality (Green et al., 2008). Even though we did not specifically investigate the reproduction of *L. terrestris* in the current experiment, the high numbers of juvenile, not identifiable earthworm species in each mesocosm indicated that hatching rates from cocoons were neither compromised by seed dressings nor the herbicide application.

When reporting non-target effects of pesticide formulations it is important to also consider side effects of numerous not-declared surfactants in these formulations as they might be more toxic than the active ingredient itself (Moore et al., 2012; Cuhra et al., 2016; Mullin et al., 2016).

### Earthworm effects

Earthworms did not interact with seed dressings or herbicide application. This is contrast to previous studies (Zaller et al., 2014, 2016; Gaupp-Berghausen et al., 2015) but might just reflect specific responses of earthworms to different pesticides. Our understanding on feedback relations between pesticides, species interactions, populations and communities is very limited and demands more detailed studies (Köhler and Triebskorn, 2013). Contrary to our expectations, earthworms reduced litter decomposition, and reduced wheat growth and biomass production. A reduction in litter decomposition by earthworms was most likely an indirect effect via physical alterations of the soil environment as the organic matter in litter bags was only accessible to soil micro- and meso-fauna but not to earthworms. Such indirect effects could result from earthworm grazing on soil fungi and microorganisms as well as on soil meso- and micro-fauna (Edwards and Fletcher, 1988; Curry and Schmidt, 2007) thereby reducing overall decomposition. Earthworms are generally considered to increase plant growth (Van Groenigen et al., 2014), but not in all situations (Zaller and Arnone, 1999) and also reduced plant growth in presence of earthworms has been observed (Zaller et al., 2013; Arnone and Zaller, 2014). However, the influence of earthworms on plant growth and biomass production, and to which direction, depend mainly on earthworm and plant species in the system (Laossi et al., 2010; Doan et al., 2013) and still much is unknown about the precise earthworm-plant relationships (Scheu, 2003) or earthworm effects on root production (Arnone and Zaller, 2014).

## Conclusions

The current findings in addition to our previous ones (Zaller et al., 2016) suggest different sensitivity of soil organisms dependent on how often pesticide treated seeds were sown. We found that micro- and meso-fauna were already influenced after a single seed dressing application, while macro-fauna responded only after the second seed dressing application in the current study. It is unclear whether this is a more widespread phenomenon because ecotoxicological tests very rarely investigate repeated applications of pesticides (Pelosi et al., 2014). To what extent pesticide-induced community tolerance is responsible for acute vs. chronic toxicity of pesticides on earthworms is another underrepresented research area. In the current study we observed for the first time interactive effects on soil organisms between pesticides in seed dressings and surface applied herbicides. This indicates that pesticide risk assessments considering a single species subjected to a one time application of one pesticide class might underestimate the real world situation in agricultural fields.

## Author contributions

JGZ, WVH conceived and designed the experiment; WVH, AT, NK, VD, JH, UP, TW, VW conducted the experiment; WVH, RK, MB, JL, AR, JGZ analyzed the data; all authors jointly wrote the manuscript.

## Funding

This study was partly funded by the Austrian Federal Ministry of Agriculture, Forestry, Environment and Water Management.

### Conflict of interest statement

The authors declare that the research was conducted in the absence of any commercial or financial relationships that could be construed as a potential conflict of interest. The reviewer AT declared a shared affiliation, though no other collaboration, with one of the authors JL to the handling Editor, who ensured that the process nevertheless met the standards of a fair and objective review.
